# Basal Autophagy Is Required for Herpes simplex Virus-2 Infection

**DOI:** 10.1038/srep12985

**Published:** 2015-08-07

**Authors:** Abraam M. Yakoub, Deepak Shukla

**Affiliations:** 1 Department of Microbiology and Immunology, University of Illinois, Chicago, IL USA, 60612; 2 Department of Ophthalmology and Visual Sciences, University of Illinois Medical Center, Chicago, IL USA, 60612

## Abstract

Autophagy is a conserved catabolic process of the cell, which plays an important role in regulating plethora of infections. The role of autophagy in Herpes simplex virus-2 (HSV-2) infection is unknown. Here, we found that HSV-2 does not allow induction of an autophagic response to infection, but maintains basal autophagy levels mostly unchanged during productive infection. Thus, we investigated the importance of basal autophagy for HSV-2 infection, using pharmacological autophagy suppression or cells genetically deficient in an autophagy-essential gene (ATG5). Interference with basal autophagy flux in cells significantly reduced viral replication and diminished the infection. These results indicate that basal autophagy plays an indispensable role required for a productive infection. Importantly, this study draws a sharp distinction between induced and basal autophagy, where the former acts as a viral clearance mechanism abrogating infection, while the latter supports infection.

Human herpesvirus-2, also known as Herpes simplex virus-2 (HSV-2), is a double-stranded DNA virus that affects over 16% of the global population aged 15–49 years, causing an immense epidemic^1^,^2^. HSV-2 primarily causes severe genital diseases associated with physical disorders (e.g. genital ulcers and inflammation) and psychosocial problems^3^,^4^. HSV-2 genital disease represents a top-alert condition due to the fact that the virus may be transmitted from mothers to neonates causing a neonatal infection with high mortality rate^5^,^6^ and the fact that HSV-2 infection is a serious risk factor for HIV infection, enhancing HIV acquisition rate by 3-4 fold^7^,^8^. Moreover, HSV-2 was also found to contribute to corneal infections and other ocular pathologies^9^,^10^, or may cause meningitis^3^,^4^,^11^.

Autophagy is a cellular catabolic pathway which degrades various cytoplasmic constituents such as misfolded proteins and protein aggregates, intracellular organelles or microbial pathogens^12^,^13^. During autophagy, the cargo to be degraded is recruited through autophagy receptors to the autophagic vesicles (autophagosomes) which eventually fuse with lysosomes, releasing their cargo that is then lysosomally degraded^12^,^13^,^14^. During conditions of nutrient deficiency, autophagy is induced to maintain energy pools in the cell, and prevent translational arrests, cell cycle delays or cell death^15^,^16^,^17^,^18^,^19^. At the basal levels, autophagy plays other important homeostatic functions such as clearance of protein aggregates, and damaged organelles^12^,^13^,^14^. Disruption of autophagy sometimes results in the failure to adapt to stress conditions or starvation and may cause death of the cell or organism under such conditions^15^,^19^.

HSV-1 and HSV-2 are closely related herpesviruses that rely on ICP34.5 protein as a major neurovirulence and neurotropism factor^20^,^21^. HSV-1 virus was shown to establish virulence via preventing launch of an autophagic response to infection, as HSV-1 ICP34.5 binds to and inhibits the autophagy mediator beclin1^22^,^23^,^24^,^25^. HSV-2 ICP34.5 shares some common features with HSV-1 ICP34.5 and also shows differences^26^,^27^,^28^. For example, HSV-2 ICP34.5 gene, contrary to HSV-1 ICP34.5, contains an intron and undergoes alternative splicing, yielding various structurally unique splice-variants^27^ and protein products^28^. However, whether HSV-2 ICP34.5 inhibits autophagy responses in cells, similarly to HSV-1 ICP34.5, remains to be established. Very little is known about the role of autophagy in HSV-2 infection. Therefore, we investigated the role of autophagy in regulating HSV-2 infection. Our results demonstrate that basal autophagy, which is maintained at stable levels during productive HSV-2 infection, is required for successful HSV-2 infection, as its disruption prevented infection to a great extent.

## Results

### Basal autophagy levels are maintained unchanged during HSV-2 infection

**Figure 1:**
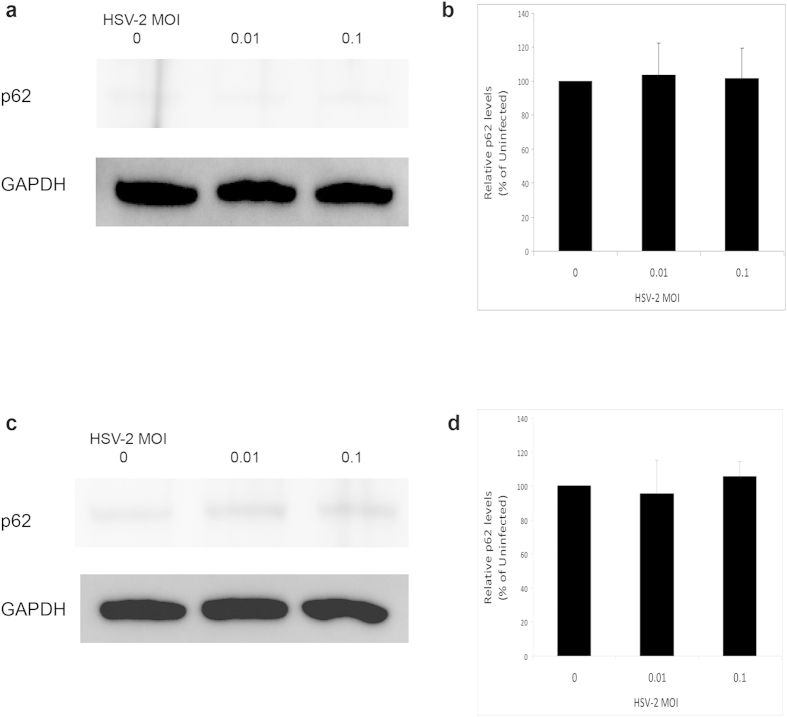
Monitoring autophagy flux during HSV-2 infection. (**a** and **c**). Human foreskin fibroblasts were uninfected or infected with different MOIs of HSV-2 for 1 hr (panel a) or 6 hrs (panels c). The cells were then harvested and lysed, and the lysate was immunoblotted for p62 to assess autophagy flux. (**b**) Quantification of the relative p62 levels in (a), after normalization to GAPDH. (**d**) Quantification of the relative p62 levels in (c), after normalization to GAPDH.

In response to HSV-1 infection, we previously found that autophagy levels may be slightly inhibited, or in most cases remain unchanged, during productive HSV-1 infection^29^. The autophagic response to HSV-2 infection was not previously assessed. Thus we monitored autophagy flux in cells during HSV-2 infection using sequestosome1 (or p62) immunoblotting. p62 is a protein that is degraded mainly by autophagy and thus its levels represent a reliable indicator of autophagy flux in cells^30^,^31^,^32^. *Id est*, accumulation of p62 marks suppression of the autophagy flux, whereas its depletion reflects autophagy induction^30^,^31^,^32^. We found that HSV-2 infection does not cause any significant changes in autophagy flux in host cells (Fig. 1a–d). This result indicated that HSV-2, similarly to HSV-1, prevents autophagy induction in response to infection, but meanwhile maintains the basal autophagy activity of the host mostly unhampered.

### Pharmacological suppression of autophagy inhibits HSV-2 infection

Having found that HSV-2 maintains the basal autophagic activity of host cells, we hypothesized that this basal autophagic activity could play a role in infection. To investigate such a role of autophagy in HSV-2 infection, we used pharmacological means to suppress cellular autophagy levels. First, we used chloroquine to suppress autophagy. We found that chloroquine treatment of the cells led to reduced viral yields in cells (data not shown). Various chemical agents have been classically used in the field to suppress autophagy, however some of these agents suffer from side effects that make it difficult to interpret whether the results are directly caused by autophagy. For example, 3-methyladenine, a commonly used autophagy inhibitor, was also reported to enhance basal autophagy despite suppressing starvation-induced autophagy^33^. In addition, chloroquine and vinblastine were shown to cause autophagy-complicating nonspecific effects such as mammalian target of rapamycin (mTOR) inhibition^34^. In a comparative study testing various side effects of autophagy inhibitors, bafilomycin A1 (BFN), a well-known autophagy inhibitor that blocks fusion of autophagosomes with lysosomes^35^, was shown to have least nonspecific effects, and no effect on mTOR activity, in treated cells in contrast to other agents tested^34^. Thus, we decided to confirm the chloroquine preliminary finding with a more specific autophagy inhibitor, BFN.

**Figure 2:**
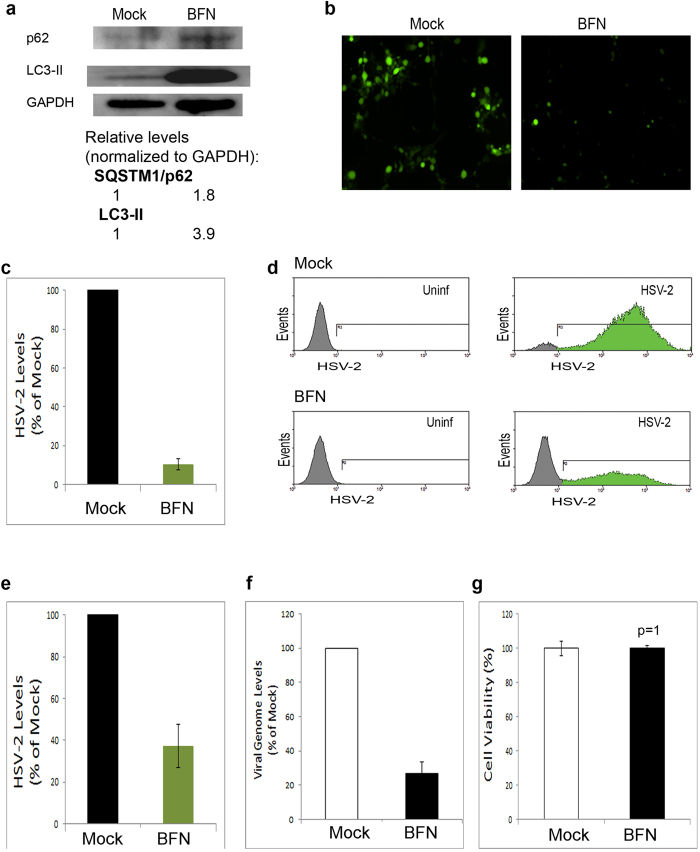
Pharmacological inhibition of autophagy suppresses HSV-2 infection. (**a**) Validation of autophagy suppression by BFN treatment: HCE cells were mock-treated or treated with BFN for 16 hours. The cells were then harvested, lysed and immunoblotted. (**b**) HCE cells were incubated with HSV-2-GFP for 2 hrs. Then, the virus was removed and the cells were replenished with medium without or with BFN. 14 hrs later, infection was monitored using fluorescence microscopy. (**c**) Quantification of relative HSV-2 levels, from experiments performed as in (b). (**d**) HCE cells were infected with HSV-2-GFP in presence or absence of BFN (as in (b)). The cells were analyzed using FACS assay at 20 hours post-infection (hpi). (**e**) Quantification of virus levels in (d), based on the percentage and mean fluorescence intensity (MFI) of the cells in the gated (green) regions of the histograms. (**f**) qPCR HSV-2 genome quantification from HSV-2-infected, mock-treated or BFN-treated, HCE cells at 16 hpi. (**g**) Cell viability under mock or BFN treatment. HCE cells were mock-, or BFN-, treated for 16 hrs. Cell death was assessed using MTT assay.

We treated human corneal epithelial (HCE) cells, known to be targeted by both HSV-1 and HSV-2 *in vivo* and *in vitro*^9^,^10^, with BFN. We then confirmed BFN-mediated suppression of autophagy through p62 and LC3 immunoblotting. Suppression of basal autophagy by BFN was confirmed by the accumulation of p62 along with LC3-II (Fig. 2a). To rule out any possible effects of BFN on viral entry, we incubated the cells with HSV-2 for 2 hrs, and the cells were then untreated or treated with BFN. Using HSV-2-GFP virus, we monitored the progress of infection microscopically. We observed a significant drop in virus levels in BFN-treated, relative to mock-treated, cells (Fig. 2b,c). To confirm this finding, we employed a quantitative FACS-based assay to measure virus yields in cells. We found that BFN-mediated block of autophagy suppressed viral yields and HSV-2 infection (Fig. 2d,e). To further confirm, we determined virus levels in the cells via qPCR virus genome quantification. qPCR assay confirmed that BFN treatment significantly reduces HSV-2 infection (Fig. 2f). Moreover, the possibility of cell death upon BFN treatment was ruled out (Fig. 2g). These results demonstrated that suppression of basal autophagy in host cells interferes with viral replication and the progress of infection.

### Monitoring HSV-2 infection under genetic deficiency of autophagy

Due to the limitations of pharmacological assays and the difficulty to completely rule out nonspecific effects of chemicals, we sought to confirm the effect of basal autophagy on HSV-2 infection using genetic means. Therefore, we used mouse embryonic fibroblasts (MEFs) that are incapable of executing autophagy due to a genetic knockout of the autophagy-essential gene ATG5^19^,^36^. Out of the 31 autophagy-related (ATG) genes, 18 genes (including ATG5 and ATG7) are essential for development and maturation of autophagosomes^37^. ATG7^−/−^ MEFs, for instance, showed reduced degradation of long-lived proteins and cytoplasmic organelles even at basal levels. These cells also exhibited impairment of autophagosome formation and maturation, and impairment of both basal and starvation-induced autophagy^38^.

**Figure 3:**
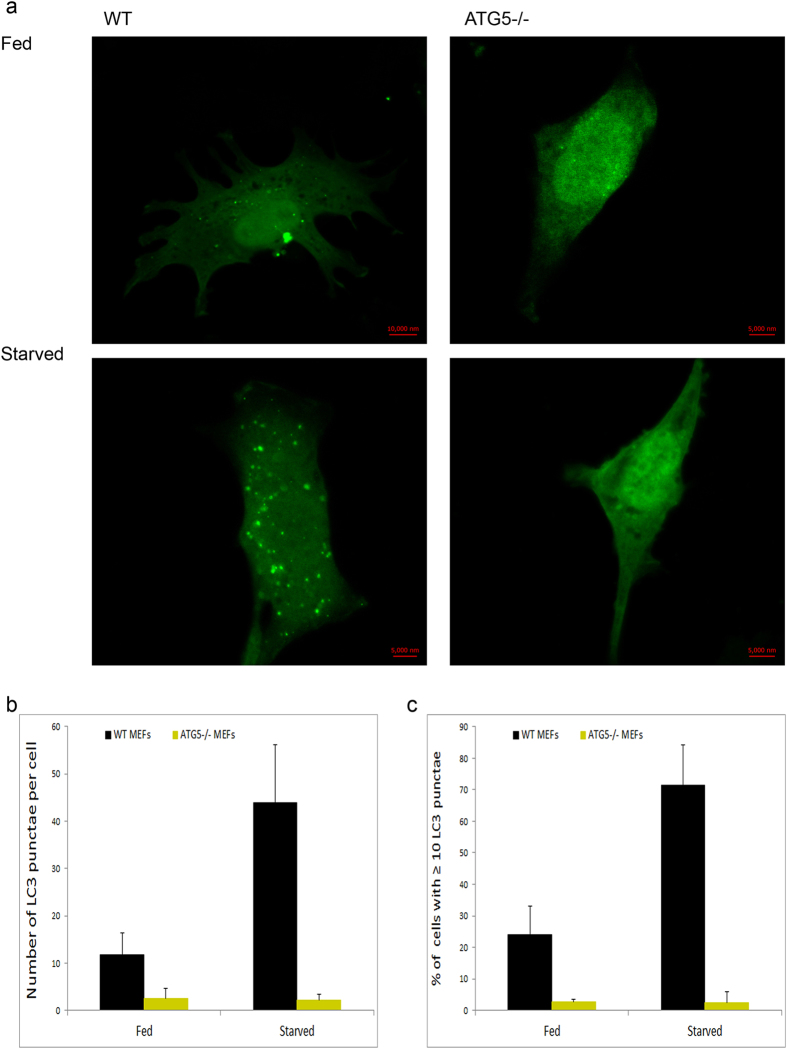
Validation of autophagy deficiency in ATG5^−/−^ cells. (**a**) LC3-GFP-expressing WT or ATG5^−/−^ MEFs were cultured in regular serum-containing medium (Fed), or starved for 3 hr. The cells were then fixed and imaged using confocal microscopy at 63x magnification. (**b**) Quantification of the number of LC3 punctae per cells, from confocal microscopy experiments performed as in (a). An average of 50 cells was assessed and used for counting. (**c**) Quantification of cells with ≥10 LC3 punctae, from confocal microscopy experiments performed as in (a). An average of 50 cells was assessed.

Before using the ATG5^−/−^ cells in our virus assays, we wanted to validate their autophagy deficiency. We monitored basal autophagosomal levels in normal feeding conditions and induced autophagosomal levels under nutrient loss (starvation). Using GFP-LC3-expressing MEFs, we were able to detect low basal levels of autophagy in wild-type (WT) cells, but much less levels in ATG5^−/−^ cells (Fig. 3a–c). In addition, ATG5^−/−^ cells were also defective in mounting an autophagic response to starvation (Fig. 3a–c). These results validated the autophagy-deficient nature of these cells.

**Figure 4:**
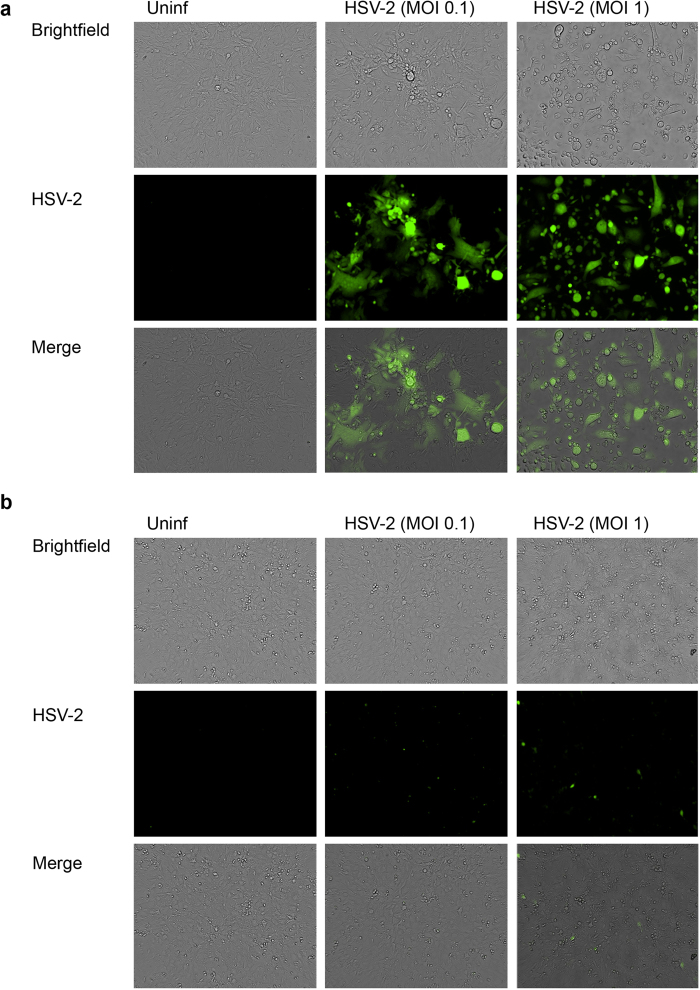
Monitoring HSV-2 infection in WT or ATG5^−/−^ cells. (**a** and **b**) WT MEFs (panel a) or ATG5^−/−^ MEFs (panel b) were uninfected or infected with HSV-2-GFP at various MOIs, and infection was monitored microscopically at 20 hpi.

Then we infected WT or ATG5^−/−^ MEFs with HSV-2-GFP at various multiplicities of infection (MOIs) and assessed the infection with fluorescence microscopy. We found that while WT MEFs showed signs of robust HSV-2 infection, including viral gene expression, viral spread, and syncytia formation (Fig. 4a), ATG5^−/−^ cells showed extremely reduced infection signs at all the MOIs tested (Fig. 4b). These findings suggested that autophagy deficiency interferes with HSV-2 infection.

### Autophagy deficiency abrogates HSV-2 infection

**Figure 5:**
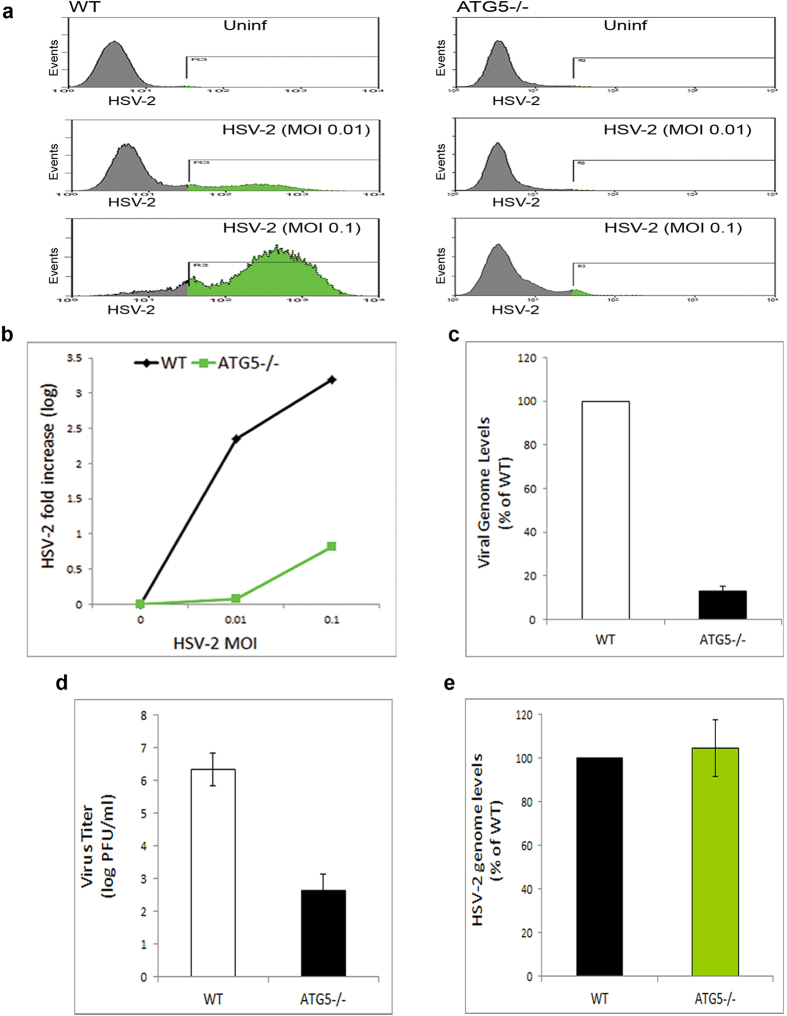
Autophagy-deficient cells are resistant to HSV-2 infection. (**a**) WT or ATG5^−/−^ MEFs were uninfected or infected with HSV-2-GFP at the indicated MOIs, and viral yields were determined by FACS analysis at 24 hpi. (**b**) Quantification of viral levels in (a), based on the integrated MFI (=percentage X MFI) of the GFP-positive population in the gated region R3. The graph shows relative fluorescence levels of infected cells to background of uninfected cells. (**c**) Viral DNA was isolated from HSV-2-infected WT or ATG5^−/−^ MEFs at 14 hpi, and quantified using qPCR. (**d**) WT or ATG5^−/−^ MEFs were infected with HSV-2. After 24 hr, the supernatants of infected cells were collected and titered using plaque assay. (**e**) WT or ATG5^−/−^ MEFs were infected with HSV-2 for 2 hrs, followed by determination of internalized virus levels using qPCR assay for viral DNA.

Having observed low infection of cells deficient in autophagy, we sought to confirm quantitatively the effect of autophagy deficiency on viral levels in infected cells. We thus utilized various quantitative assays of infection: FACS assay, qPCR viral genome quantification and virus titer determination by plaque assay. Compared to WT cells, ATG5^−/−^ cells showed significantly lower viral yields and seemed highly resistant to infection (Fig. 5a,b). Even at higher MOIs, ATG5^−/−^ MEFs still showed significant block of infection (Fig. 5a,b). qPCR assay also showed that ATG5 knockout in cells dramatically diminishes the infection (Fig. 5c). Finally, we determined virus titer from WT or ATG5^−/−^ cells using plaque assay. While WT cells secreted approximately 10^2^-10^3^ plaque-forming units (PFU)/monolayer, ATG5^−/−^ cells secreted approximately 10^6^-10^7^ PFU/monolayer (Fig. 5d). Additionally, in order to rule out the possibility that autophagy deficiency may interfere with virus entry into cells, we assessed internalized virus levels in WT or ATG5^−/−^ cells. We found that autophagy deficiency does not affect virus entry into the cells (Fig. 5e). Together, our results indicate that basal autophagic activity of the host cells supports HSV-2 infection, which is consistent with results of pharmacological autophagy inhibition (Fig. 2).

### In-depth validation of the role of basal autophagy in HSV-2 infection

**Figure 6:**
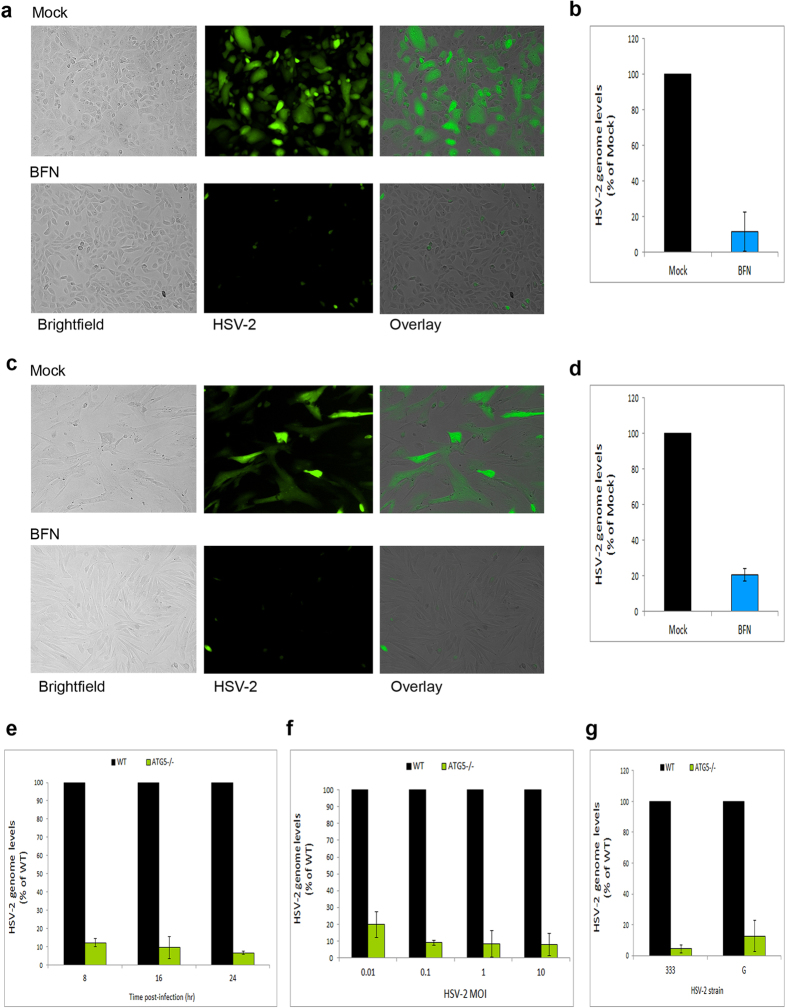
In-depth assessment of HSV-2 productive infection under autophagy inhibition or deficiency conditions. (**a**) ARPE19 cells were infected with HSV-2-GFP for 2 hrs, and then replenished with medium with or without BFN. Infection was monitored microscopically at 12 hpi. (**b**) ARPE19 cells were infected with HSV-2 for 2 hrs, followed by mock- or BFN-treatment. At 16 hpi, intracellular viral genomes were determined by qPCR. (**c**) Human foreskin fibroblasts were infected with HSV-2-GFP for 2 hrs, followed by mock- or BFN-treatment. Infection was monitored microscopically at 16 hpi. (**d**) Human foreskin fibroblasts were infected with HSV-2 for 2 hrs, followed by mock- or BFN-treatment. At 20 hpi, intracellular viral genomes were determined by qPCR. (**e)** WT or ATG5^−/−^ MEFs were infected with HSV-2 for the indicated durations. At each time point, viral replication was assessed by determining viral DNA genomes by qPCR. (**f**) WT or ATG5^−/−^ MEFs were infected with various MOIs of HSV-2. At 16 hpi, viral genomes were quantified by qPCR. (**g**) WT or ATG5^−/−^ MEFs were infected with HSV-2 strains 333 or G for 16 hrs. Infection was then monitored via intracellular viral genome determination by qPCR.

We then sought to evaluate the role of autophagy in HSV-2 infection under various productive infection conditions and in multiple infection systems. First, we tested the effect of autophagy inhibition on infection in other cell types. We found that autophagy suppression caused a significant reduction of infection in a retinal pigment epithelial cell line (Fig. 6a,b) or in human foreskin fibroblast cells (Fig. 6c,d). Second, using ATG5^−/−^ cells, we assessed the importance of autophagy for infection at various time points and under infection with various MOIs. We found that autophagy deficiency was always associated with diminished viral replication levels and infection under all these condition (Fig. 6e,f). Finally, this requirement of autophagy for infection was independent of the virus strain used (Fig. 6g). Collectively, these results confirm that basal autophagy is an important factor required for a robust HSV-2 infection.

## Discussion

Autophagy plays a pivotal role as a quality control mechanism and in regulating infections and coordinating the cellular response to a pathogen attack. Induction of autophagy was previously shown to enhance HSV-1 viral clearance and diminish viral growth^23^,^39^. HSV-1 establishes virulence through inhibiting cellular autophagy^22^,^23^, or at least preventing its activation^29^, during infection. In this study, we show that HSV-2 prevents autophagy induction but maintains the basal levels of the pathway (Fig. 1). Although reports suggested that HSV-1 may inhibit autophagy via ICP34.5-mediated beclin1 binding while HSV-2 ICP34.5 was not tested for its ability to bind beclin1, our results may suggest that HSV-2 ICP34.5 also binds beclin1 to a certain extent; however this hypothesis remains to be experimentally investigated. Notably, a closely related α-herpesvirus, varicella-zoster virus (VZV), which lacks the ICP34.5 gene can allow robust induction of autophagy responses to infection^40^,^41^. Moreover, in similarity to HSV-2 results shown here, autophagy suppression downregulated VZV virus replication and growth in cells^40^. Thus, these findings together suggest that these different viruses may benefit from the autophagy pathway to varying extents and perhaps the viral autophagy-modulating genes serve to optimize or fine-tune the autophagy flux to levels that bolster each virus’s growth, according to the differential reliance of each virus on autophagy. The present study provides insights that may support this concept which requires further work to be firmly established.

Two modes of autophagy exist under different conditions, which play distinct functions^37^,^42^. Under certain autophagy-inducing conditions such as nutrient deficiency and some viral infections, autophagy may be induced above its constitutive levels. Induced autophagy qualifies the cell to adapt to drastic conditions and generate required energy from alternative sources, or to combat infections^12^,^13^,^14^,^39^,^42^,^43^. On the other hand, basal autophagy performs various homeostatic and quality control functions such as preventing protein aggregation or limiting reactive oxygen species (ROS) production^37^,^38^. Basal autophagy takes place at low or modest levels under normal conditions and regardless of the feeding conditions, as in embryogenesis^44^. A prominent example of basal autophagy is autophagy in neuronal cells and the brain. Autophagy in the brain proceeds at its basal levels, without significant induction even under nutritional deficiency (due to the fact that the brain can quickly compensate nutrient deficiency by relying on nutritional supply from other organs). Disruption of such basal autophagy leads to neurodegeneration, indicating that the role of basal autophagy is important for normal neuronal cell functioning and survival^45^. Moreover, suckling and non-suckling ATG7^−/−^ mice all died at the same time, within 1 day of birth^38^, suggesting that the reason of death is not due to nutrient deprivation and that it may be due to disruption of a cellular function performed by basal autophagy. The absence of induced autophagy in HSV-1 and HSV-2 infection and persistence of basal autophagy suggest a role for this basal autophagy that supports infection (as we show here for HSV-2).

For possible reasons for the contribution of basal autophagy to infection, some scenarios may be proposed based on lessons learned from other viruses. First, the virus may in fact be protected in the autophagosomes from more severe innate immune defense mechanisms^43^. Second, autophagosomal inclusion of the virus might support a step in the viral life cycle such as using the vesicle membranes for viral envelopment^46^,^47^,^48^. Third, autophagic vesicles may facilitate intracellular trafficking of the virions. For example, virions can be released from the cell via fusion of the lysosomes with the plasma membrane^46^. Fourth, autophagy performs many homeostatic functions, which might support infection. Future work is to determine the exact mechanism by which constitutive autophagic activity (or machinery) supports HSV infection.

Another possibility for the reason of resistance of ATG5^−/−^ cells to HSV-2 infection may be attributed to an aberrant proinflammatory cytokine response to infection in these cells. For example, HSV-1 blocks NALP3 (or NLRP3) inflammasome activity during the course of infection^49^. Inflammasomes induce maturation and secretion of proinflammatory cytokines^50^, which represent powerful antiviral effectors^51^,^52^,^53^. Autophagy-deficient cells (lacking LC3B or beclin1) showed increased NALP3 inflammasome activity and caspase-1 levels, and exhibited enhanced inflammatory responses (e.g. interleukin (IL)-1β, and IL-18) to lipopolysaccharide *in vitro* and *in vivo*^54^. Autophagy could also limit inflammasome activity directly through targeting inflammatory cytokines such as IL-1β for autolysosomal degradation^55^. Additionally, several autophagy proteins target components of the antiviral type-I interferon (IFN) response. Atg5–Atg12 blocks caspase recruitment domain (CARD)-mediated signaling and the downstream production of type I IFN^56^,^57^. Also, Atg9 was shown to regulate STING (Stimulator of Interferon Genes) cytoplasmic localization and thus control IFN-I responses^58^,^59^. It is thus possible to hypothesize that autophagy-deficient cells inherently possess elevated cytokine levels that help confer on these cells resistance to HSV-2 infection.

In addition to the role of autophagy in regulating an innate immune response to infection, housekeeping functions of autophagy may also provide a possible reason for supporting HSV-2 infection. First, autophagy-mediated removal of damaged organelles such as mitochondria (mitophagy) prevents accumulation of reactive oxygen species (ROS) (Ref. 60). ROS may induce NALP3 inflammasomes^61^, type-I IFN responses^62^, or cytokine production in response to infection^63^. Compounds that enhance ROS production suppress HSV infection^64^, while factors that relieve herpesvirus-induced oxidative stress^65^ significantly enhance infection^66^,^67^. Second, protein aggregation under autophagy-deficient conditions^38^ could also present a reason for infection halt. Third, dysregulation, in ATG5^−/−^ cells, of metabolism and turnover of metabolites known to regulate infection^68^,^69^ may contribute to their resistance to HSV-2 infection. Finally, a distinct role of autophagy machinery in enodosomal trafficking and endolysosomal functions was recently reported^70^. Perhaps, impairment in autophagy-deficient cells of intracellular endosomal trafficking of viral, or infection-regulating cellular, components participates to infection suppression in these cells.

In conclusion, this study demonstrates the importance of basal autophagy in HSV-2 infection. Induced and basal autophagy may serve distinct functions, and their dysregulation causes morbidities and mortalities^19^,^71^,^72^ and influences susceptibility to various pathogens^73^,^74^. Importantly, this study draws a difference between induced and basal autophagy in HSV infections, and will incite future mechanistic studies of the basal autophagy-mediated support of herpesvirus infection.

## Methods

### Cells and cell culture

Human corneal epithelial (HCE) cells were a kind gift from K. Hayashi (National Eye Institute, Bethesda, MD). HCE cells were cultured in Minimum Essential Medium (MEM; Gibco) supplemented with penicillin/streptomycin (Gibco) and 10% fetal bovine serum (FBS; Sigma). Mouse embryonic fibroblasts (MEFs) were kindly provided by C-A A. Hu (University of New Mexico, Albuquerque, NM). Human retinal pigment epithelial cell line (ARPE19), human foreskin fibroblasts, and African green monkey foetal kidney epithelial (Vero) cells were provided by H. Ying (University of Illinois, Chicago, IL), N. Luraine (Rush University, Chicago, IL) and P. Spear (Northwestern University, Chicago, IL), respectively. MEFs, ARPE19, human foreskin fibroblasts and Vero cells were grown in Dulbecco Modified Eagle’s Medium (DMEM; Gibco) supplemented with antibiotics and serum.

### Viruses

HSV-2 (strain 333) and HSV-2 (333)-GFP viruses were provided by J. Vieira (University of Washington). HSV-2 strain G was provided by P. Spear (Northwestern University, Chicago, IL). Viruses were propagated on Vero cells according to standard procedures.

### Antibodies

Anti-LC3 polyclonal antibodies were from Novus Biologicals (Catalog number NB100-2220). Anti-GAPDH and anti-SQSTM1/p62 polyclonal antibodies were purchased from Santa Cruz (Catalog number sc-25778 and sc-25575, respectively). Horseradish peroxidase-conjugated secondary (anti-rabbit) antibodies were purchased from Jackson Immunoresearch (Catalog number 111-005-144).

### Infection

The cells were incubated with the virus in phosphate-buffered saline (PBS) supplemented with 0.1% glucose and 1% serum at 37 °C-5% CO_2_ conditions. After 2 hrs, the virus was removed and fresh medium was added to the cells.

### Immunoblotting

The cells were harvested, and lysed in RIPA buffer (Sigma, Catalog number R0278) containing protease-phosphatase inhibitors. Cell lysates were electrophoresed on denaturing SDS-PAGE gel (Novex), and proteins were transferred onto a PVDF membrane, followed by blocking of non-specific binding, incubation with primary antibody and horse radish peroxidase (HRP)-conjugated secondary antibody. The membrane was developed by incubation with Femto-Sensitivity ECL (Thermo), and bands were imaged using ImageQuant LAS4000 digital imager (GE).

### Pharmacological inhibition of autophagy

For inhibition of autophagy, Bafilomycin A1 (LC Labora-tories, Catalog number 118 B-1080) at 100 nM concentration was used.

### Fluorescence microscopy

The cells were washed in PBS, and imaged using Axiovert 100 M fluorescence microscope (Zeiss). Image acquisition and analysis were performed using MetaMorph software (Zeiss).

### Flow cytometry

After infection, the cells were washed in FACS buffer (PBS, 1% BSA, 0.05% NaN_3_), and analyzed cytofluorimetrically on LSRFortessa cytometer (BD). Analysis of FACS data was performed using Summit software (Beckman Coulter).

### Viral genome isolation and quantification

Cell pellets were re-suspended in buffer containing 1% SDS, 50 mM Tris (pH 7.5), and 10 mM EDTA, and the cell extract was incubated with proteinase K (50 μg/mL) at 37 °C for 1 hr. DNA was then extracted by phenol/chloroform extraction-ethanol precipitation procedure. Viral DNA was quantified via quantitative PCR (qPCR) on an ABI 7500 Fast thermocycler (Applied Biosystems), using HSV-specific primers (Forward 5′-TAC AAC CTG ACC ATC GCT TG-3′, Reverse 5′-GCC CCC AGA GAC TTG TTG TA-3′) which amplify the glycoprotein D (gD) gene of HSV-2.

### Plasmids

pEX-GFP-hLC3WT plasmid (referred to as GFP-LC3) previously described^75^ was obtained from Addgene (plasmid #24987).

### Transfection

Transfection was performed using Lipofectamine 2000 (Invuitrogen) according to the manufacturer’s guidelines.

### Starvation

To induce autophagy by starvation, the cells were washed in PBS for three times to remove residual medium, and then were cultured in Hanks’ Balanced Salt Solution (Gibco) for 2-3 hr.

### Confocal fluorescence microscopy

To monitor LC3-GFP punctae, confocal microscopy (Zeiss 710 microscope, Zeiss) was used. After treatment, the cells were washed, fixed in paraformaldehyde, and used in imaging. Image acquisition was performed using ZEN software (Zeiss), and image analysis was performed using MetaMorph software (Zeiss).

### Virus titer (plaque formation) Assay

Confluent Vero cell monolayers were infected with serially diluted virus for 2 hrs. Then the cells were washed and covered with methylcellulose (Sigma)-containing DMEM. The cells were grown for 72 hrs, then fixed and stained with crystal violet. Plaques were counted, and plaque counts were used to calculate viral titers.

### Cell viability (MTT) assay

Cell viability was assessed using MTT (3-(4,5-Dimethyl-2-thiazolyl)-2,5-diphenyl-2H-tetrazolium bromide) assay. MTT was purchased from Sigma. The assay was performed as previously described^76^.

### Statistical analyses

Experiments were independently replicated for at least three times. Quantification figures show mean values, and error bars represent standard error of the mean. Statistical significance was determined via Student’s t-test (minimum p-value for significance 0.05). Unless otherwise indicated, the data were statistically significant (p-values < 0.05).

## Additional Information

**How to cite this article**: Yakoub, A. M. and Shukla, D. Basal Autophagy Is Required for Herpes simplex Virus-2 Infection. *Sci. Rep*. **5**, 12985; doi: 10.1038/srep12985 (2015).
